# Clinical Reports—Medical Wards of Mercy Hospital

**Published:** 1870-11

**Authors:** 


### Clinical Reports.

### CLINICAL REPORTS—MEDICAL WARDS OF MERCY HOSPITAL.

Clinic of PROF. DAVIS. Reported by S———.

*Oct. 13, 1870.*—Case 1st, DIABETES MELLITUS.

Male; aged 40 years; native of Ireland; laborer; has been affected with symptoms of diabetes about one year. Shortly previous to the appearance of these symptoms, he had two attacks of throwing up blood to the extent of one pint or more at a time. Whether the blood came from the lungs or stomach is not very easily determined by the account given by the patient. He also suffered, at the same time, from loss of appetite and indigestion. The evacuations from the bowels have generally been regular—once or twice a-day.

During the last two or three months, his appetite has been good, thirst excessive, and yet he has steadily declined in flesh and strength, is morbidly sensitive to atmospheric changes, and voids from one to two gallons of urine per day. (Here the attention of the class was directed to two large glass-jars, filled with light straw-colored urine, which was the quantity he had passed during the last twelve hours.) At present, the patient's skin is harsh, rough, and dry, especially on the hands and neck; his pulse is soft, weak, and a little increased in frequency; moderately emaciated; muscular weakness; and mentally despondent. The taste of the urine present is sweet, and its odor characteristic. It readily enters into fermentation by the yeast test. (Their attention was directed to a specimen showing the process of fermentation in progress, and Dr. Lyman Ware, one of the physicians to the Dispensary, kindly exhibited a specimen of the fermenting urine under the microscope, affording each student a good view of the torulæ, or fungi, developed by fermentation.)

Trommer's, and other tests, might be applied, but the foregoing, in connection with the general symptoms, are sufficient to render the diagnosis certain in this case.

In commenting on the case, the Professor said, substantially, that the early symptoms of diabetes are generally obscure, and that the disease is often associated with tuberculosis. The digestive function is generally impaired; for, though the appetite may be good, or even voracious, yet digestion is slow, and accompanied by fulness, eructations, and mental depression. The bowels are inactive, the skin dry, and a gradually increasing thirst. As the disease progresses, the dry and harsh condition of the skin, and the loss of flesh and strength become more manifest, and the patient often complains of weariness and some pains in the back, and, sometimes, sharp neuralgic pains in different parts of his system. The existence of these symptoms, and especially the steady loss of flesh and strength, while the patient is eating and drinking, as well as urinating more than natural, should always cause us to suspect the existence of diabetes. And if, with these symptoms, the application of the proper tests shows the presence of sugar, the diagnosis is rendered certain. A mere excessive secretion of urine, however, is not sufficient to indicate saccharine diabetes. In certain paroxysms of nervous excitement, the kidneys will secrete double or tripple the natural quantity of urine in a given time; but, in such cases, the urine is limpid, like spring water, and of low specific gravity, while in diabetes mellitus the specific gravity is high. In the case before us, the specific gravity of the urine, when the man first came under treatment, was 1040.

In albuminuria the urine is generally of high specific gravity; but here the *quantity* is generally small, and the skin and countenance of the patient is pale and bloated, instead of corrugated and shrunken.

The tendency of diabetes, when left to itself, is to steadily increase until the patient becomes fatally exhausted; but its progress is generally slow. In many cases, the symptoms are improved during the warm months of summer, and become aggravated by the cold and damp of autumn and winter. An atmosphere that relaxes the skin and increases the activity of its function, seems to lessen the activity of the kidneys, and thereby ameliorates the condition of the patient.

The pathology of the disease is still involved in obscurity. It was long since ascertained that the excessive quantity and saccharine quality of the urine did not depend on any specific or characteristic lesion of the kidneys. These organs appear to be only the outlet for an excessive quantity of glucose or saccharine matter, that finds its way into the blood from some other source; but from what other source is not yet fully determined. The experiments of Bernard and of Drs. A. Flint, Sr. and Jr., seemed to establish the fact that the liver was an active sugar-producing organ, and hence it became probable that derangement of the functions of this organ constituted the true source of the diabetes. But still later experiments have, at least, rendered it doubtful whether the sugar detected in the liver is not from *post mortem* changes.

It has long been known that somewhere in the processes of digestion and assimilation, the starch and gum taken as food become converted into sugar; and, in health, the sugar is further converted into lactic acids and other products. But, in diabetes, this further change fails to take place, and the sugar remains in excess in the blood, stimulating the kidneys to excessive action, and, consequently, maintaining a constant drain upon the whole system.

TREATMENT.—The indications for the treatment of diabetes are, first, to exclude, as far as possible, all elements of food capable of being converted into sugar in the process of digestion. Second, to so alter the processes of digestion and assimilation as to complete the conversion of the saccharine products of digestion into lactic acid, or such other constituents as are capable of being appropriated to the tissues, or excreted without disturbing the function of the kidneys. To accomplish these purposes, there is probably no more efficient method than to keep the patient steadily on the use of skimmed milk, buttermilk, or milk-whey, bran-bread, and lean meat. For, fresh vegetables, onions, and cabbages may be allowed, as they contain but little starch or sugar. As an occasional change, a porridge, made of roasted or scorched cornmeal, may be allowed. In addition to the regulation of diet, I have found much advantage in the use of a teaspoonful of the liquid Bennett immediately after each meal. In some instances, a pill, containing pulv. opii half a grain, and sulph. cupri one-sixth of a grain, taken before breakfast and dinner, will, also, lessen the quantity of urine.

The wearing of flannel next the skin, and a warm bath two or three times a week, are, also, valuable aids. Diabetes is a very obstinate, and often incurable, disease; yet, I have seen a few cases cured by a persevering use of the foregoing means.

